# Developing an Immersive Virtual Reality Training System for Novel Pediatric Power Wheelchair Users: Protocol for a Feasibility Study

**DOI:** 10.2196/39140

**Published:** 2022-10-06

**Authors:** Sara Drisdelle, Liam Power, Scott Thieu, Jordan Sheriko

**Affiliations:** 1 Department of Pediatrics Izaak Walton Killam Health Centre Halifax, NS Canada; 2 Faculty of Medicine Dalhousie University Halifax, NS Canada

**Keywords:** immersive virtual reality, power wheelchair, training, pediatric rehabilitation, feasibility

## Abstract

**Background:**

Power wheelchairs can empower children with physical limitations to gain independence in their everyday lives; however, traditional methods of power wheelchair training are often limited by poor accessibility and safety concerns. Immersive virtual reality technology (IVRT) uses advanced display technology to place users in a fully immersive web-based environment that can support real-time skills training, often requiring less resources and fewer safety concerns than real-world methods. IVRT interventions have shown to be a feasible training option among adult power wheelchair users; however, there is still a need to understand the technical and clinical feasibility of developing an IVRT power wheelchair training tool for the pediatric population.

**Objective:**

This proposed study aims to use expert feedback and an iterative design process to develop an IVRT training intervention for pediatric power wheelchair skill development.

**Methods:**

This 3-phase feasibility study will be conducted within the assistive technology unit of a public pediatric hospital. Separate participant groups will be recruited for each phase, consisting of approximately 10 to 15 clinicians (phase 1), 10 pediatric power wheelchair users (phase 2), and 15 to 20 additional pediatric power wheelchair users (phase 3). Phase 1 will be conducted to gather feedback on the baseline IVRT training intervention. Clinicians will test the intervention and assess its usability and acceptability using qualitative and quantitative methods. Phase 1 participants will also be invited back for a subsequent session to reassess a revised version of the training intervention that has been updated based on their previous feedback. Phase 2 and phase 3 will also use mixed methods to gather feedback on the usability, acceptability, and user experience of the IVRT training intervention from current pediatric power wheelchair users. In addition, phase 3 participants will perform a skills transfer assessment to compare power mobility skill performance between the virtual reality and real-life environments. Data gathered in phase 2 will be used to further refine the IVRT intervention, whereas phase 3 data will be used to statistically evaluate the final version.

**Results:**

This study was approved by the Izaak Walton Killam Health Centre research ethics board in August 2021. Phase 1 testing began in February 2022. The entire study is expected to be completed by 2023.

**Conclusions:**

The results of this study will be used to create an IVRT training intervention for pediatric power wheelchair skill development through an iterative and collaborative design process. Results may also assist in directing future studies in this area.

**International Registered Report Identifier (IRRID):**

DERR1-10.2196/39140

## Introduction

### Background

The inability to mobilize independently can have significant deleterious effects on the psychosocial development of children, often affecting their ability to participate in age-relevant activities [1,2]. For children with physical disabilities that limit their ability to ambulate, assistive devices such as power wheelchairs empower them to mobilize with independence and create great opportunities for play, leading to enhanced intrapersonal and interpersonal relationships [2-7]. Children who use power wheelchairs describe feeling liberated and gaining autonomy with powered mobility use, with some even reporting the device as an integral part of their identity and an extension of the self [6]. Despite the benefits of power wheelchair use, methods of its training can be highly variable and difficult to access [8]. Many children are unable to participate in training opportunities owing to common barriers such as limited access, safety concerns, and inadequate availability of resources [5,6]. Furthermore, the rate of skill acquisition is often not a linear process, and it can be difficult for training interventions to account for individual differences among clients, including skill level and age range [5-8]. Access to power wheelchair training opportunities is essential to develop the skills required for independent participation and overall development [1,2]. As such, there exists a need for an approach that offers effective training for novel power wheelchair users within a safe and accessible environment.

### Virtual Reality for Rehabilitation

Virtual reality (VR) is emerging as a promising modality for therapeutic and rehabilitative interventions in health care [9,10]. VR interventions have been shown to help support the rehabilitative process, by improving functional and cognitive performance in diverse populations such as patients with Parkinson disease [11], patients with chronic stroke [12], older adults with cognitive impairment [13], and power wheelchair users [14]. The growing interest in VR may be owing to the unique opportunity that allows individuals to engage in task-specific interventions by interacting in a real-time simulation of a computer-controlled activity or environment [9,10,15]. The use of a computer interface can also allow clinicians to quickly adjust the intervention to suit the user’s current ability (eg, modification of the difficulty level), thus promoting user awareness and confidence [16,17].

Children who use a VR application to practice their power wheelchair skills have shown increased overall improvement when transferring their skills into real-world navigation compared with their pretraining scores or a control group with no training [16,18-20]. A study examining the efficacy of a desktop VR system in teaching novel power wheelchair skills found that the VR intervention improved children’s skills to a greater extent than the control group who had no VR training; however, statistical significance was not reached [20]. In this case, individual performance scores varied greatly owing to potential confounders (eg, sex differences and potential motivation discrepancies), and it was suggested that future VR interventions should consider individualizing their training methods to meet the varying needs and interests of participants [20].

### Immersive VR Technology

Recent VR modalities such as immersive VR technology (IVRT) may elicit increased performance improvements over time compared with nonimmersive VR systems owing to an increase in user engagement [21-24]. IVRT is a specific subset of VR that uses display technology such as head-mounted display (HMD) goggles or multiple screen projections to make the user feel physically present in a 3D setting [25]. The use of screen projections to create a fully immersive environment can be resource-intensive, often requiring a large space and multiple pieces of technological equipment to achieve a realistic setting. In contrast, HMD technology requires less costly resources and small physical space and can be easily transported to allow for clinical or at-home use [26,27]. Therefore, IVRT interventions using HMD tend to be the primary choice for recent skills-based training applications [28].

One of the greatest benefits of IVRT is the sense of presence that can be experienced by users during gameplay. The feeling of truly *being there* in the VR environment is heightened within an immersive VR system compared with a nonimmersive system and has been linked to better performance [21-24]. In a 2017 study comparing wheelchair performance and visual technology devices, the sense of presence and driving performance were both increased among users who trained with the IVRT modality compared with those who trained with a computer monitor [21]. Studies have also shown that users participating in an IVRT simulator are able to naturally mimic the same wheelchair-specific movement patterns (eg, trunk posture and chair propulsion) as executed in the real world, thus demonstrating the feeling of realism that can be experienced in the immersive environment [29].

IVRT has shown promise as a feasible rehabilitation tool for power wheelchair users; however, most studies have been conducted only among the adult population [14]. A 2019 scoping review found that most published studies using HMD-based IVRT for power wheelchair simulation included only adult participants, whereas a limited number of studies have extended into the pediatric population [14,16,30]. Morère et al [30] used a 3D wheelchair simulator to conduct a chronic training intervention and identified a positive change in pediatric participants’ outdoor driving abilities after completion of the training period; however, this study included only 12 participants in total. Another pediatric study revealed improvements in real-world power wheelchair skills following training with HMD compared with pretraining levels, but this study was also limited in sample size [16].

IVRT has the potential to become a valuable training tool for pediatric power wheelchair users, but there is a paucity of literature on this topic, with weak descriptions of methodology and limited sample populations [8,31]. Introducing IVRT training for pediatric power wheelchair users may help to enhance opportunities for safe and accessible skill development, leading to increased independence and improved early-life psychosocial development [2-7]. To develop an effective method of power wheelchair training, there exists a need for collaborative studies in which expert-driven feedback can be used to design a training intervention that meets the needs of pediatric power wheelchair users.

### Objectives and Research Questions

#### Overview

This proposed 3-phase feasibility study will collect feedback from experienced clinicians and pediatric power wheelchair users to collaboratively develop an HMD-based IVRT training platform designed for pediatric power wheelchair skill development. Participants will engage in the training intervention and provide feedback on the usability and acceptability of the intervention for novel skill development. In this study, usability refers to the ease with which participants can successfully engage in the IVRT training intervention [32]. Feedback related to usability will assist in identifying features of the intervention that may help to achieve specific goals easily and effectively with limited confusion during gameplay. Acceptability is the perceived appropriateness of the intervention to meet the needs of the target population (novel pediatric power wheelchair users) [33]. Acceptability feedback will describe features of the IVRT training intervention that may help to enhance clinical uptake and accurately capture the training requirements of the pediatric population. Clinicians will also assist in developing a list of potential power wheelchair skills to be targeted in the training intervention.

Feedback gathered during each of the 3 phases will be carefully implemented in the technical design to continuously refine the training platform and produce a final version that can be used as a practical training tool for effective skill development in the future [34]. The efficacy of the final IVRT training intervention for power wheelchair skills training will be measured in future studies. To the best of our knowledge, this is the first study as of April 2022 that will gather iterative feedback from clinicians and experienced power wheelchair users to develop an IVRT training intervention intended for pediatric power wheelchair skill development.

#### Objective

##### Overview

To determine the feasibility of using an IVRT training intervention for pediatric power wheelchair skill development, as determined by the following:

1. Expert opinion from clinicians experienced in working with pediatric power wheelchair users
2. User feedback and skill performance metrics gathered from current pediatric power wheelchair users

##### Research Question 1

What is the usability of the IVRT training intervention for novel skill development, from the clinician’s and current power wheelchair user’s perspectives?

##### Research Question 2

What is the acceptability of the IVRT training intervention for pediatric power wheelchair users, from the clinician’s and current power wheelchair user’s perspectives?

##### Research Question 3

What set of skills should be included in the IVRT training intervention for appropriate power wheelchair skill development, from the clinician’s perspective?

We hypothesized that iterative feedback gathered from clinicians and current power wheelchair users will create an IVRT training intervention that is appropriate for our target population and can be successfully used for future power wheelchair skill development.

## Methods

### Study Design

This is a 3-phase feasibility study that will assess the usability and acceptability of an IVRT training intervention that has been collaboratively designed to support power wheelchair skill development. Mixed methods will be applied to provide qualitative and quantitative data on the outcome measures.

### Study Setting

This study will be conducted within the assistive technology unit of a public pediatric hospital, the Izaak Walton Killam Health Centre in Halifax, Nova Scotia, Canada. An experienced researcher will facilitate all in-laboratory IVRT sessions and collect training intervention data. All power wheelchair skills performed in the IVRT training system and in the real world during phase 3 will be independently assessed by a clinician trained in power wheelchair skills assessment.

### Participants and Recruitment

Eligible participants for this feasibility study will belong to one of 2 population groups. The first population group will consist of clinicians with at least three months of experience in working with individuals who use power wheelchairs (by means of training or offering care services) and who practice in a health care profession such as physiotherapists, occupational therapists, or child life specialists. The second population group will consist of children (aged 4-18 years) who currently use power wheelchairs. The lower age limit is selected to ensure that the VR headset will properly fit all participants, whereas the upper limit is representative of the pediatric population. A complete list of inclusion and exclusion criteria is provided in Textbox 1.

For phase 1, our target sample size is approximately 10 to 15 participants, consistent with common sample sizes used in technology feasibility studies with an iterative design process [35-37]. The target sample size for phase 2 will be 10 participants, to gather user experience data and facilitate 1 round of iterative development based on user feedback. For phase 3, we aim to recruit 15 to 20 participants for the purpose of statistically assessing the final version of the IVRT training intervention through user feedback and skill transferability. The target sample size for phase 3 is consistent with large studies piloting non-VR power wheelchair training methodologies [38,39] and is also greater than that used in previous pediatric IVRT feasibility studies [16,30].

Clinicians will be recruited for phase 1 via web-based advertisements (email lists and web-based newsletters) and word of mouth. Pediatric participants will be recruited for phase 2 and phase 3 via web-based newsletters, poster advertisements displayed within the host hospital, and discussion with their care provider. Researchers will distribute study information forms to care providers, which will outline the study design, purpose, and contact information. Care providers will be encouraged to offer these forms to their patients if it is believed that they may be interested in participating. Then, the individuals will indicate their interest to a research team member via email or verbally, and eligible individuals will be invited to participate in the study.

**Inclusion and exclusion criteria for each participant group.**

**Inclusion criteria for clinicians (phase 1)**

- Overall, ≥3 months of experience in working with power wheelchair users
- Practices in a health care setting
- Able to communicate fluently in English (verbal and writing)
- Able to operate a standard power wheelchair joystick

**Exclusion criteria for clinicians (phase 1)**

- History or suspicion of a photosensitive seizure disorder
- Unable to tolerate wearing head-mounted display goggles for prolonged periods of time
- Impairment in visual functioning that cannot be corrected with lenses or contacts (eg, 3D depth perception, cataracts, and oculomotor dysfunction)

**Inclusion criteria for current power wheelchair users (phases 2 and 3)**

- Aged 4-18 years
- Current power wheelchair user, with ≥1 year of experience in using power wheelchair as primary means of mobility
- Able to communicate verbally in English
- Able to operate a standard power wheelchair joystick

**Exclusion criteria for current power wheelchair users (phases 2 and 3)**

- History or suspicion of a photosensitive seizure disorder
- Unable to tolerate wearing head-mounted display goggles for prolonged periods of time
- Impairment in visual functioning that cannot be corrected with lenses or contacts (eg, 3D depth perception, cataracts, and oculomotor dysfunction)
- Participated in phase 2 of this study (phase 3 participants only)

### Ethics Approval

This study has been approved by the Izaak Walton Killam Health Centre research ethics board (Office of Research Ethics 1026934) in Halifax, Nova Scotia, Canada. Informed consent will be obtained from all study participants before their participation; participants aged <18 years will complete the assent form, and participants aged ≥18 years will complete the consent form.

### Procedure

#### IVRT Equipment

**Figure 1:**
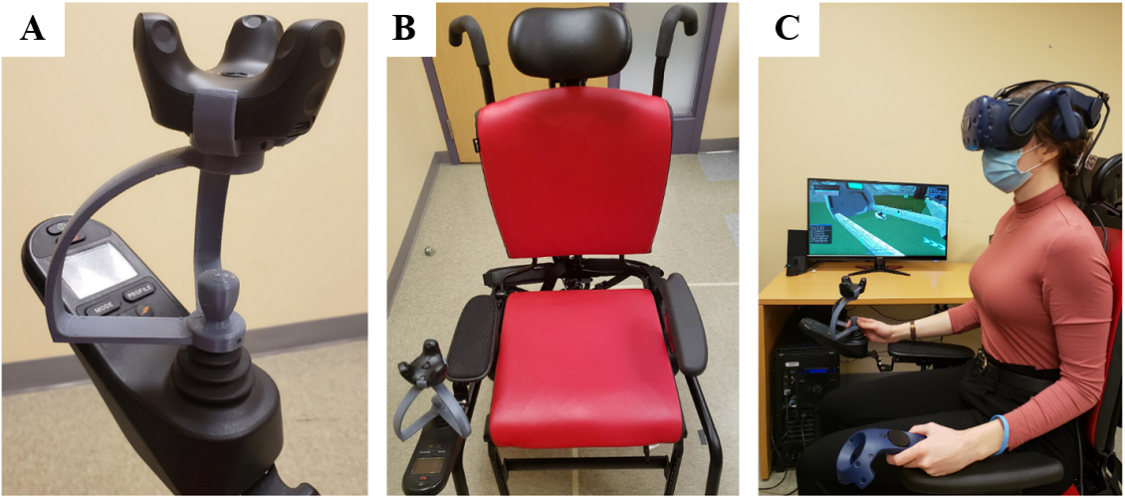
An HTC Corporation tracker and IVRT joystick (A) attached to the Rifton Activity Chair (B) and in-use during gameplay of the training intervention (C). IVRT: immersive virtual reality technology.

The IVRT training application has been built using Unity3D, a Unity Technologies game engine that allows developers to create and manage web-based gaming environments. Participants will engage in the IVRT training intervention using the HTC Vive Pro, a commercially available VR technology from HTC Corporation that places users in a fully immersive VR environment using the HMD headset, tracking devices, and controllers (Figure 1) [40]. The wheelchair joystick used for this study has been built by an engineering team using a 3D printer and specifications that closely match a real-world power wheelchair joystick. An HTC Vive Pro tracking device is attached to the top of the joystick to allow for accurate control of the power wheelchair within the IVRT environment (Figure 1). Henceforth, this joystick will be referred to as the “tracker joystick.” The user can also engage in hand-based activities (eg, turning on the power wheelchair) in the IVRT environment by moving a controller held in their nondominant hand.

In phase 1, participants will sit in a Rifton Equipment Activity Chair [41] that replicates the seating and joystick setup of a power wheelchair. The Activity Chair is a positioning chair that provides adaptable seating for a range of patient populations and has been slightly modified to accommodate an arm attachment for easy operation of the tracker joystick (Figure 1). In phase 2 and phase 3, participants will sit in their own power wheelchair, and the tracker joystick will be attached to their personal joystick controller stem. The tracker joystick is fitted to easily screw onto any standard-size joystick controller stem, allowing for users to participate in the IVRT intervention without the need to transfer to a new chair. During the IVRT simulation, users will progress through the intervention in a power wheelchair that replicates the natural movement of a real power wheelchair. The in-game power wheelchair movement patterns (eg, acceleration and deceleration speeds and turning trajectories) have been designed in collaboration with an occupational therapist to ensure that all movements are simulated with accuracy.

#### IVRT Intervention

The IVRT training intervention has been designed to help users develop core power wheelchair skills in a motivating and engaging manner. The baseline version of the training intervention was created in collaboration with the research committee (consisting of power wheelchair rehabilitation experts, including a pediatric physiatrist and a pediatric occupational therapist) and software development team. The research committee identified power wheelchair tasks that were commonly taught to novel power wheelchair users as beginner to moderate–level skills (eg, moving forward, moving backward, and turning 90°) [42]. Then, the software development team integrated the skills into the IVRT system within an environment that was approved by the research team to be acceptable for the pediatric population (ie, containing age-appropriate graphics and characters). The baseline training intervention is intended to introduce participants to the potential of an IVRT system for pediatric power wheelchair training and gather expert feedback on how to improve the skills, environment, and overall user experience.

**Figure 2:**
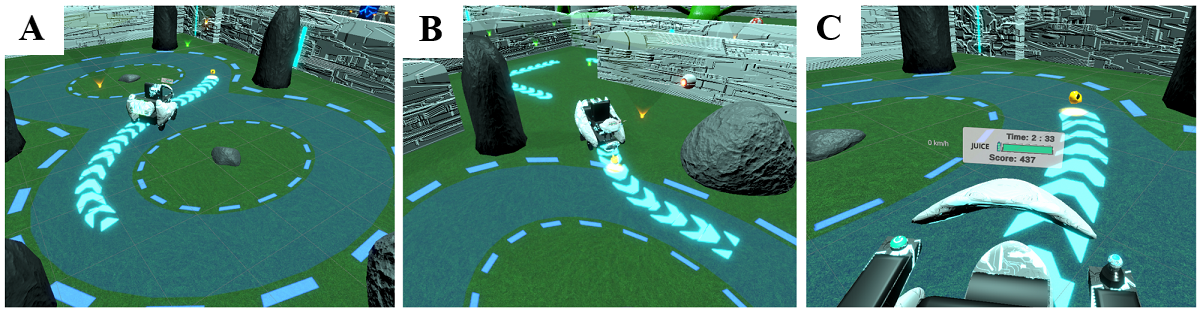
Screenshots of the training application showing stages of the figure-eight task from three different camera viewpoints: follow camera (A), free camera (B), and first-person view (C).

The intervention places participants in a colorful cartoon environment with robotic characters. Users begin the intervention in the main lobby, where the instructor of the game explains the instructions and wheelchair controls using audio and visual cues. Then, the participants are brought to the outside world to begin level 1, where they must use the tracker joystick to control their power wheelchair and move through specific areas in the game to complete each level. Users will be challenged to participate in tasks that integrate various power wheelchair skills, such as backing up in a narrow hallway, driving over a ramp, and following a figure-eight path (Figure 2). In addition, players are also provided with various opportunities to increase their scores during gameplay, including driving on the correct path and collecting fruits to power up their wheelchair. The inclusion of a reward system that allows for positive reinforcement has been shown to enhance a child’s attention and motivation when training within a VR environment [43-45]. Therefore, it is anticipated that these additional components will help to increase user engagement and improve performance outcomes.

The IVRT system offers a range of options to measure client performance, which will otherwise be difficult to capture in real-world training. In total, 4 different VR camera angles (overhead, follow camera, first-person view, and free camera) provide the operator with a variety of viewpoints to assess the user’s driving skills (Figure 2). Chart selections can also be accessed by the operator to evaluate in-game user behavior, such as *client focus* (measurement of attentional focus) and *pathways* (real-time charting of user’s driving patterns). Furthermore, client skills and goals can be easily tracked using the metrics options, which offer real-time speed, collision, and completion time statistics. Taken together, the IVRT system can provide an accurate and comprehensive assessment of driving performance without disrupting the in-game user experience.

#### Training Intervention—Phase 1

The aim of phase 1 is 2-fold: (1) to use clinician feedback to develop a list of potential power mobility skills to be implemented in future versions of the IVRT training intervention and (2) to assess the usability and acceptability of the intervention from the clinician’s perspective. Phase 1 participants, comprising health care clinicians, will be exposed to a baseline version of the IVRT training intervention and participate in a training session during which they will progress through each of the levels, with instructions to carefully evaluate various components of the intervention. Participants will also be given the option to freely explore the game environment following the completion of each level, if they wish to do so.

Participants will be asked to assess the ability of the intervention to teach a range of core power wheelchair skills. Currently, there are no standardized methods that have been established among professionals to assess power mobility skills among children [38]; however, 4 main measures are commonly used: Assessment of Learning Powered Mobility Use [46], Powered Mobility Program [47], Power Mobility Training Tool [48], and Wheelchair Skills Checklist [49]. In the adult population, the Wheelchair Skills Test for Powered Wheelchairs is well validated for the evaluation of power wheelchair capacity [42]. Owing to the lack of an established measurement tool for the pediatric population, the research team identified core skills frequently listed in both the Wheelchair Skills Test for Powered Wheelchairs and common pediatric assessment tools to create a comprehensive skills list for clinician use. This list was developed in consultation with a pediatric rehabilitation specialist to ensure that all skills were appropriate for inclusion.

Immediately following the IVRT training intervention, participants will be provided with a list of 28 individual power mobility skills and asked to indicate their level of agreement with the following statement: “Based on my experience working with power wheelchair users, I believe the immersive virtual reality technology (IVRT) application can be used to teach children and adolescents to (insert skill here)*.”* Consensus on the application’s ability to assess each item will be defined as ≥75% of participants indicating that they agree (score=4 out of 5) or strongly agree (score=5 out of 5) for a given item, leading to the inclusion of the skill in subsequent phases. If ≥75% of participants indicate disagreement (score=2 out of 5) or strong disagreement (score=1 out of 5) for a particular item, this will be deemed as consensus that the intervention is not appropriate for the development of that skill, and it will not be included in future phases. If a neutral response (score=3 out of 5) is indicated by ≥75% of participants, the skill will be refined for future versions of the training intervention using participant feedback. If an item is rated <4 out of 5, participants will be asked to provide a specific recommendation for modification of the IVRT intervention pertaining to that skill. If a participant believes that a skill cannot be feasibly modified, they will provide no response in the recommendation section.

Participants will also be provided with 3 separate questionnaires to assess perceived usability, acceptability, and overall user experience. Quantitative data will be collected from (1) the Presence Questionnaire (PQ) [50], (2) an ad hoc usability and acceptability questionnaire, and (3) an ad hoc user experience questionnaire. Qualitative data will be collected from (1) an ad hoc user experience questionnaire; (2) participants’ informal in-game comments and reports, as recorded by the researcher; and (3) a semistructured interview (round 2 only). A complete list of the assessment measures included in each phase is provided in Table 1.

The PQ and ad hoc usability and acceptability questionnaire will be provided as paper-based materials. The PQ is a well-validated assessment tool used to measure presence within a VR environment. In this study, the PQ has been adapted to include the 4 subscales most relevant to the IVRT training intervention: realism, possibility to act, quality of interface, and self-evaluation of performance [50]. Participants will be asked to report their experience related to multiple components of each subscale using a 7-point Likert scale rating. The ad hoc usability and acceptability questionnaire has been adapted from the Perceived Usefulness and Perceived Ease of Use scales [51] and System Usability Scale [52]. This questionnaire will be used to explore participants’ attitudes toward using the IVRT intervention as a training tool for the pediatric population. Participants will be asked to rank each statement on the questionnaire from 1 to 5, ranging from “strongly agree” to “strongly disagree.”

**Table 1:** Assessments by phase and round.

Assessments	Phase 1 (clinicians)			Phase 2 (power wheelchair users)		Phase 3 (power wheelchair users)
	Round 1	Round 2	Round 1		Round 1	
Power wheelchair skills inclusion checklist	✓^a^					
Presence Questionnaire	✓	✓	✓		✓	
Usability and acceptability questionnaire	✓	✓	✓		✓	
User experience questionnaire	✓	✓	✓		✓	
Semistructured user experience interview		✓	✓		✓	
Training methods questionnaire and interview (power wheelchair user and caregiver)			✓		✓	
Skill transferability assessment					✓	

^a^✓: the check mark specifies the assessment measures that will be used in each phase and round of the study.

The ad hoc user experience questionnaire will be hosted on the secure web-based software platform, REDCap (Research Electronic Data Capture; Vanderbilt University) [53]. The web-based questionnaire format was chosen to allow participants to respond to open-ended questions by typing rather than writing; however, they will be provided the option to complete a paper-based questionnaire if it is preferred. This questionnaire will be used to assess participant demographics, user tolerance (a component of usability), and overall user experience. To capture user tolerance to the IVRT intervention, the presence of VR-induced symptoms and effects (VRISE) will be assessed during and after the intervention. VRISE includes symptoms such as nausea, dizziness, disorientation, and fatigue and can occur as a side effect of VR exposure [51]. VR systems using HMD have been found to increase the prevalence of VRISE compared with nonimmersive systems; however, our intervention’s length falls below the theoretical limit of exposure to VR for adults (55-70 minutes) [54,55]. Although our intervention session is approximately 20 to 30 minutes in length, it is anticipated that some participants may still experience VRISE symptoms. User experience will be assessed in this questionnaire using open-ended questions that have been developed to better understand participants’ experiences within the IVRT application, such as ease of use, appropriateness of tasks and graphics, and suggestions for improvement.

Following the completion of the study session, participant feedback data will be summarized, anonymized, and presented to the research committee. The committee will use these data to produce recommendations for a new iteration of the application, which will be implemented by the software development team.

Participants will be invited back to the laboratory to complete a second session during which they will engage in the IVRT training intervention that has been updated based on feedback from the round-1 sessions. We aim to have at least 60% (ie, >7 out of 12 participants) of phase 1 participants return for second round of testing, based on previous health care studies that have received a similar percentage of participant retention for multiround testing [56,57]. The second round of testing is intended to check for accuracy and ensure that participants are satisfied with the changes implemented based on feedback from the first session. Participants will complete the updated IVRT training intervention, followed by a semistructured interview designed to gather in-depth details of the user experience. The interview questions will be developed based on data from round 1 and will aim to capture feedback regarding the system’s new updates (eg, opinions regarding any new skills or levels added and updated graphics or audio) and address any potential areas for further improvement. Finally, participants will also complete the same PQ, ad hoc usability and acceptability questionnaire, and ad hoc user experience questionnaire as in round 1. Participant feedback will be presented to the research committee, and if any items in the IVRT intervention are found to still require significant changes, they will be updated as necessary by the software development team.

#### Training Intervention—Phase 2

Phase 2 will assess the usability and acceptability of the IVRT system from the pediatric power wheelchair user’s perspective. Participants in phase 2 will be comprised of current pediatric power wheelchair users, who will test and evaluate the updated IVRT training system that has been adjusted based on feedback gathered from clinicians in phase 1.

During the IVRT trial, participants will be placed in the IVRT setup and provided with 5 to 10 minutes to freely explore and acclimate to the VR setting. Once the participants indicate that they are ready to begin, they will start the training intervention. The skills selected in phase 1 for inclusion will be integrated into the intervention, and participants will be encouraged to complete each skill as they progress through the levels. Following completion of the intervention, participants will be given the option to exit the system or continue exploring after a mandatory 10-minute break. After the break, participants may freely explore the VR environment for up to an additional 15 minutes at their own discretion. The mandatory 10-minute break has been included in the session to reduce consistent VR exposure and limit the potential of VRISE among children [58].

Immediately following the IVRT intervention, participants will complete the same 3 questionnaires as in phase 1, but with age-appropriate adaptations (eg, changes to wording or question structure). Age-appropriate adaptations will be approved by a child life specialist to ensure suitability for the pediatric population. All questionnaires will be asked aloud by the researcher, and the pediatric participant’s verbal responses will be recorded. As in phase 1, the questionnaires will explore the perceived usability and acceptability of the IVRT system for power wheelchair skill development, IVRT tolerability, and general user experience.

Participants will also engage in a semistructured user experience interview, in which questions will be asked aloud and responses will be audio-recorded for qualitative analysis. The semistructured interview intends to gather in-depth details on the perceived usability, acceptability, and experience in the IVRT environment (eg, most favorite and least favorite parts of the game and why and areas for improvement). The participant’s caregiver (parent or proxy) will also be encouraged to provide any additional details that the pediatric participant may not remember (eg, dates and early-life experiences).

Pediatric participants and their caregiver will also participate in a training methods questionnaire and interview during the session. The training methods questionnaire will use Likert scale questions (asked aloud by the researcher) to explore both the child’s and caregiver’s perceptions of previous power wheelchair training methods. Then, a semistructured interview will be conducted to further explore their experience with power wheelchair training (eg, most exciting or challenging parts of training and confidence in skills after training). This information will provide great understanding of past training techniques and experiences from 2 different perspectives. In all cases where pediatric participants cannot remember specific details, caregiver input will be sought to ensure completeness of the data set.

After all participants have completed their session, data will be collected, summarized, and presented in the same manner as in phase 1. The research committee will use this feedback to identify and implement changes to the application for the final phase.

#### Training Intervention—Phase 3

Phase 3 of this study will also assess the usability and acceptability of the IVRT system from the perspective of current pediatric power wheelchair users. In addition, participants will complete a real-world trial and an IVRT trial to compare power mobility skill transfer between the VR and real-life environments. Both trials will occur over 1 study session, and the order of trials will be counterbalanced among participants.

All phase 3 participants will test the IVRT training system that has been updated based on phase 1 and phase 2 feedback. To compare and assess participant’s skill transferability, an experienced clinician will review in-game and real-world performance. It is anticipated that the IVRT intervention will be designed to closely resemble a real-life setting; therefore, the skills transferability assessment will measure the similarity of participant’s skills performance across both the VR and real-life environments.

A computerized recording of the IVRT intervention will be independently assessed by the clinician following the completion of the session to compare in-application versus real-world performance metrics. During the real-world trial, participants will be asked to complete each skill that has been included in the IVRT intervention. Skills will be performed in an environment within the hospital grounds and assessed by an experienced clinician. In-game and real-life performance will be assessed for capacity level (skill performed: “yes” or “no” and skill proficiency rating from 0-3) and time to complete each skill.

Participants will also be asked to complete the same questionnaires and semistructured user experience interview as in phase 2 to explore their experience in the IVRT environment and the perceived usability and acceptability of the intervention. Caregivers will again be encouraged to assist in the user experience interview to provide further details as needed and to complete the same ad hoc training methods questionnaire and interview as in phase 2. Exploratory analyses are planned to be conducted with phase 3 data to further evaluate the final version of the IVRT intervention.

### Risk to Participants

Previous studies have shown that IVRT can be administered to children and adults without inducing significant safety risks to participants [54,55,58]. The research team will actively monitor for the presence of VRISE or any signs of discomfort during exposure and provide medical follow-up as necessary. The risk of physical injury to pediatric participants is low and will be mitigated by including only experienced power wheelchair users and conducting the real-world skills assessment in a large open space within hospital grounds.

### Primary Outcomes

#### Acceptability

Acceptability of the skills targeted in the IVRT training intervention will be defined and assessed by clinicians using a core power wheelchair skills list. A structured web-based survey will be presented to clinicians following the completion of the IVRT training intervention, outlining 28 potential skills for inclusion. Participants will be asked to indicate their rating for each of the listed skills using a 5-point Likert scale ranging from “strongly agree” to “strongly disagree” for inclusion. Respondents will also have the option to provide skill modification recommendations in an open text field.

Acceptability of the IVRT training intervention will be assessed in each of the phases using 3 separate questionnaires: the PQ, an ad hoc usability and acceptability questionnaire, and an ad hoc user experience questionnaire. The questionnaires will consist of questions from the same topic for each phase; however, the wording of the questions will be adjusted to suit individual participant groups. Quantitative data will be collected using the questionnaires to investigate the perceived effectiveness of the intervention, suitability for the pediatric population, and sense of presence. Qualitative data will further explore users’ attitudes and experiences with IVRT. Open-ended question prompts in the first round of phase 1 (eg, perceived safety, appropriateness of graphics, and areas for improvement) will further evaluate the suitability of the intervention for our target population, whereas semistructured interview responses will capture in-depth qualitative data to check for accuracy and validity related to the updated IVRT intervention.

#### Usability

Usability of the IVRT training intervention will be determined through the ad hoc usability and acceptability questionnaire, ad hoc user experience questionnaire, and skills transfer assessment (phase 3 only). Phase 3 participants will complete the same skills in their real-world power wheelchair as those in the IVRT intervention and will be assessed by an experienced clinician to compare wheelchair skill performance. In doing so, skill transferability between the VR and real-world environments can be analyzed to identify similarities or discrepancies in user performance across environments. It is anticipated that this study will not be powered to detect any clinically significant discrepancies in skill transferability; however, data will be used to explore IVRT versus real-world skill transfer.

Participants will complete the ad hoc usability and acceptability questionnaire and user experience questionnaire to explore their attitudes toward the system’s effectiveness and complexity. Quantitative data will define perceived ease of use, user confidence, and level of satisfaction, whereas qualitative data will help to identify potential barriers or facilitators in using the intervention, such as presence of in-game confusion, uncertainty of tasks, and particular areas of frustration or excitement. Tolerability of the IVRT intervention will be assessed during gameplay and through a VRISE section on the user experience questionnaire to determine any symptoms experienced during or after the intervention. Informal notes taken by the researcher will also record comments or questions asked by the user during the intervention to further measure usability.

### Secondary Outcomes

Pediatric participants and their caregivers will be asked to describe their previous experience with power wheelchair training methods and perceived abilities of the power wheelchair user. A semistructured interview and Likert scale questions will inquire about the location, length, and satisfaction of previous wheelchair training methods; confidence in abilities; and support received or challenges faced with training. Responses will be used to further understand the experiences with power wheelchair training from both the pediatric user and caregiver perspectives and integrate this information into future iterations of the IVRT system.

### Statistical Analysis Plan

The data analysis plan for phase 1 focuses on descriptive and thematic analyses to compare the usability and acceptability of the IVRT intervention across participants and to create a list of potential power wheelchair skills for inclusion in subsequent phases. Phase 2 analysis will focus on descriptive and thematic analyses to understand user experience via system usability, acceptability, and previous training data. Phase 3 will use a descriptive and thematic analysis approach similar to phase 2 to examine system usability, acceptability, user experience, and descriptive and interferential statistics to assess performance outcomes and compare skill transferability from the real world to the IVRT intervention.

This study will define consensus based on clinical suggestions from Nair et al [59]. Phase 1 consensus for the inclusion of items in the power mobility skills list will be reached if a minimum of 75% (9/12) of participants agree (score=4 out of 5) or strongly agree (score=5 out of 5) on a given skill. Skills that do not achieve consensus will not be included in the IVRT intervention for future phases. The inclusion of skills in the phase 2 version of the IVRT intervention will be based on the perceived usefulness and necessity of the skill (as determined by phase 1 participants) and technical feasibility of integrating the skill into the IVRT environment (as determined by the software developers). Descriptive statistics will also be calculated for each skill item and presented to the panel members as feedback in phase 1.

All questionnaire responses to closed questions and Likert-type rating scales will be analyzed using RStudio. In phase 3, real-world and IVRT skill performance data will use “completed” or “not completed” scores and ratings of capacity from 0 to 3 to assess participants’ performance outcomes for each specific skill. Then, a composite score will be created and included in the descriptive analysis to compare the performance metrics across participants and environments. In phase 3, inferential statistical testing will also be conducted to evaluate skill transferability; it is anticipated that Wilcoxon signed-rank test will be used to compare real-world and in-application performance data among participants.

Open-ended questionnaire and semistructured interview responses will be thematically analyzed using NVivo (version 12; QSR International) [60]. Questions have been developed in consultation with a pediatric rehabilitation specialist to ensure clarity and appropriateness for each population group. The semistructured interview questions in each phase will be developed to reflect topics of interest (eg, highly variable responses or recurring topics) that arise from the initial feedback round and subsequent rounds. Results will be formatted in a document file and presented to the research panel after the completion of each round.

## Results

Institutional review board approval was received in August 2021, and recruitment for phase 1 of this study began in February 2022. As of September 2022, a total of 12 participants enrolled in round 1 and 5 (42%) participants returned for round 2. Phase 1 is expected to be completed in October 2022.

Preliminary data analysis was conducted on phase 1–round 1 data in June 2022. Qualitative data explored the clinician’s user experience and revealed a positive perception of the IVRT system as a feasible tool for the pediatric population; however, adjustments in the system’s graphics and audio were suggested to reduce overstimulation, complex language, and nausea. Quantitative data further supported the clinical usability of the system and determined potential skills for inclusion into future versions. It is expected that full analysis for phase 1 data will begin in October 2022.

Phase 2 and phase 3 are anticipated to begin in fall of 2022 and winter of 2023, respectively, and it is expected that the entire study will be completed by summer 2023. Results are planned to be published in a peer-reviewed journal in early 2024 and used to develop a future research trial that will test the efficacy of the IVRT training intervention.

## Discussion

### Overview

Providing pediatric power wheelchair users with adequate skills training is fundamental when looking to improve their independence and well-being [1,2]. Unfortunately, many children are often restricted from accessing traditional training opportunities owing to physical and environmental barriers [5,6]. Without the knowledge of basic power mobility skills, children with physical disabilities are often unable to participate in activities that help to foster long-term social and cognitive development [1,2]. Therefore, it is essential to create an avenue through which children can develop power mobility skills in a manner that is accessible, easy to use, and safe.

VR is an innovative technique that can create expansive power wheelchair training opportunities within a setting that is often safer and less resource-intensive than a traditional training environment. HMD-based IVRT offers significant training benefits owing to the system’s fully immersive components and low resource requirements [26-28]. Although IVRT is beginning to emerge as a potential approach to power mobility training for children, current studies are still in their infancy [8,31]. This study will use the knowledge of multiple expert groups to collaboratively assess and design an IVRT training system that can be used for power wheelchair skill development in the future.

The IVRT application developed through this project will be deliberately designed for engaging pediatric populations; however, by assessing core power wheelchair skills relevant to novel users of all ages, future versions of the IVRT training system may be tailored for a variety of populations. In the future, we also hope to integrate machine learning engines into IVRT technology. These engines will apply real-time data output to generate specific tasks and graphics that can be tailored to match the individual needs of each user.

The short-term goal of this study is to develop an IVRT training intervention that has high usability and acceptability ratings among clinicians and pediatric power wheelchair users. The long-term goal is to provide novel power wheelchair users with a high-quality clinical training intervention that can be easily accessed to safely develop their power wheelchair skills. We also anticipate that the findings from this study will contribute to enhancing the current knowledge on IVRT for clinical practices, as IVRT is currently an underused technology that has the potential to improve patient outcomes by increasing user motivation [15,16], reducing resource requirements [26,27], and enhancing opportunities for task individualization [42-44].

### Strengths and Limitations

A main strength of this study lies within the involvement of multiple participant groups to collectively critique and develop the IVRT training intervention. Although it is more time intensive to include both pediatric power wheelchair users and clinician participant groups, collecting data from people across various professions, ages, and life experiences will ensure that diverse opinions can be integrated into the development process. In addition, the use of consensus testing in this study will ensure that the final skills chosen for inclusion in the training intervention are relevant to our pediatric population and approved by experienced clinicians. The use of a mixed methods technique also strengthens our study by providing a detailed understanding of the perspectives, barriers to, and facilitators of IVRT skills training, as recognized by each participant group. Mixed methods can be a particularly useful tool when working in disability and rehabilitation research, as it uses multiple techniques to capture information for both population-based and individual analysis that may be otherwise missed when using only one structured method [61,62]. Given the novelty of our IVRT training intervention, it is particularly important to understand user experience from various angles to develop a final product that has been assessed by multiple expert groups.

A primary weakness of this study is the potential homogeneity of participants’ attitudes toward the use of technology for rehabilitative purposes. It is possible that individuals who decline to participate in the study may have differing opinions on the usefulness or acceptability of IVRT compared with participants who agree to participate. We aim to mitigate this potential bias by intentionally recruiting participants with a range of demographics (eg, age and profession) to gather diverse perspectives. The limited sample size in this study will also affect the generalizability of the findings and underpower any inferential statistical analysis performed. Therefore, statistical analyses will be interpreted with caution and used to inform future development of the IVRT intervention rather than to define any conclusive results. Similarly, our pediatric sample is limited by the exclusion of participants who are nonverbal. To obtain comprehensive user feedback in this study, all participants must be able to communicate verbally; however, we hope to include both participants who are verbal and those who are nonverbal in future IVRT studies to gather training data that can be generalized to both population groups. Finally, the visually immersive quality of the IVRT intervention may create feelings of fatigue or motion sickness among users, possibly affecting in-game user performance or postgame assessment measures. The IVRT application is equipped with antinausea settings that can be added to the user’s visual field to help reduce VRISE. Aspects of VRISE will also be measured after exposure to identify any distress that may be further alleviated and minimized in future versions.

### Conclusions

This proposed feasibility study aims to develop and assess an HMD-based IVRT training intervention intended to benefit children with mobility limitations by creating a safe and accessible means of power wheelchair skill development. Exploring the acceptability and usability of the intervention is the first step in creating a final version that may be further tested in future studies and eventually implemented as part of clinical practices in rehabilitation health care. Given the limited number of pediatric studies using HMD-based IVRT for power wheelchair training currently published [16,30], findings from this study may also be used to inform the methodology, study procedures, and assessment protocol of future large-scale IVRT trials.

## Abbreviations

HMD: head-mounted display
IVRT: immersive virtual reality technology
IWK: Izaak Walton Killam
PQ: Presence Questionnaire
REDCap: Research Electronic Data Capture
VR: virtual reality
VRISE: virtual reality–induced symptoms and effects

## Data Availability

Data sharing is not applicable to this paper, as no complete data sets were generated at this stage of the study. However, upon completion of this study, the data generated and analyzed will be available from the corresponding author (SD) upon reasonable request. Results are also expected to be published in a peer-reviewed journal.
